# Standardizing sterilization practices for surgical implants: insights from the Asia Safe Surgical Implant Consortium

**DOI:** 10.1017/ash.2026.10377

**Published:** 2026-06-18

**Authors:** Wing Hong Seto, Patricia Ching, Anucha Apisarnthanarak

**Affiliations:** 1 The University of Hong Kong School of Public Health, Hong Kong; 2 Hong Kong Adventist Hospital, Hong Kong; 3 Thammasat University Hospital, Thailand

## Abstract

**Objective:**

Hospital-acquired infections (HAIs) pose significant challenges in the Asia Pacific region, where disparities in healthcare infrastructure and inconsistent infection prevention protocols may exacerbate patient safety risks. A recent survey of healthcare facilities across ten Asia-Pacific countries identified substantial gaps in compliance with international guidelines for sterilizing surgical implants, indicating a need for educational initiatives to drive improvement.

**Methods:**

The Asia Safe Surgical Implant Consortium was established through collaboration among regional professional associations and met in Tokyo, Japan, to formulate consensus recommendations.

**Results:**

The consensus process incorporated diverse expert perspectives and current evidence, resulting in three foundational documents that addressed quality assurance in sterilization, standardized management of loaner and implantable devices, and the implementation of a robust sterilization recall policy. These recommendations emphasize the integrated use of physical, chemical, and biological indicators, thorough documentation, and timely communication to enhance sterilization practices. Ongoing regional collaboration, investment in resources, and professional development were identified as critical for achieving consistent adoption of best practices and improving surgical outcomes.

**Conclusions:**

The consortium’s work provides a structured framework to support central sterile supply departments and elevate patient safety standards across the Asia Pacific region.

## Commentary

Hospital-acquired infections (HAIs) are a major global concern, leading to extended treatment times, increased healthcare costs, and higher mortality rates. The burden of HAIs is particularly significant in the Asia Pacific region due to a combination of factors such as high population density, disparities in healthcare infrastructure, and the complexities involved in implementing effective infection prevention across diverse healthcare settings. The effectiveness of HAI prevention efforts is further hindered by inconsistencies in surveillance systems and reporting protocols, which often result in gaps in data collection and may contribute to the underreporting of infection rates.^1–3^ These deficits not only obscure the true scale of the problem but also impede the development of targeted interventions. Within this context, the proper sterilization of medical devices emerges as a fundamental basis for safeguarding patient safety and minimizing the risk of HAIs.^4^ Medical devices that are not properly sterilized can transmit harmful microorganisms, leading to infections in patients. The monitoring of sterilization practices is crucial to maintaining high safety standards and ensuring strict adherence to established protocols, thereby promoting better health outcomes across the region.

## Advancing Asia sterilization practices for surgical implants

In 2023, a comprehensive survey evaluated the sterilization practices for surgical implants at 521 healthcare facilities across ten countries in the Western Pacific and Southeast Asia.^5^ The study focused on assessing the extent to which these practices conformed to established international guidelines and identifying factors affecting compliance. The results revealed significant adherence gaps, including improperly prepared loads for reprocessing, insufficient monitoring, and deviations from protocol driven by operational pressures. Many respondents did not monitor implant loads with both process challenge devices (PCD) and indicators, released loads before biological indicator (BI) results, lacked chemical indicator (CI) guides, and relied heavily on immediate use steam sterilization (IUSS), especially for loaner instruments. The survey revealed a need for regional collaboration to produce consensus documents and educational programs to develop standardized practices for implant-load monitoring and IUSS practices. Therefore, the authors concluded that establishing a consortium to launch educational initiatives for Southeast Asia and Western Pacific countries would be beneficial.

The Asia Safe Surgical Implant Consortium aimed to address current sterilization guidelines and develop consensus recommendations that will promote the standardization of practices across central sterile supply departments (CSSD) throughout the Asia Pacific region.

## Formation of the Asia Safe Surgical Implant Consortium

The World Health Organization (WHO) Collaborating Center, School of Public Health of the University of Hong Kong established the Asia Safe Surgical Implant Consortium with the WHO and prominent regional professional associations: Asia Pacific Society of Infection Control, Australian College of Perioperative Nurses, Central Sterilizing Services Association of Thailand, Federation of Sterilizing Research and Advisory Councils of Australia, Perkumpulan Praktisi Sentral Sterilisasi Indonesia, Japan periOperative Nursing Academy, Korea Association of Central Supply Department Nurses, Korean Association of Operating Room Nurses, Malaysian Sterile Service Association, New Zealand Sterile Sciences Association, Singapore Infection Control Association, Singapore Operating Room Nurses Chapter, Thai Perioperative Nurses Association, Viet Nam Infection Control Society, and World Federation for Hospital Sterilization Sciences. Each association nominated representatives to join the consortium, resulting in the attendance of 20 delegates at the meeting. Each association was requested to submit its latest standards and guidelines, policies, and procedures related to quality assurance in sterilization, recall procedure, and management of implant and loaner instruments. The submission of these associations including WHO guidelines on decontamination and reprocessing of medical devices for health-care facilities were distributed to the delegates prior to the meeting, with preliminary feedback requested via email.

The meeting convened on June 23, 2023, in Tokyo, Japan, where a comprehensive review of key standards and guidelines pertaining to sterilization quality assurance across the Asia Pacific region was undertaken. Particular emphasis was placed on best practices in sterilization, infection prevention strategies, and the utilization of physical, chemical, and biological indicators. Policies regarding recall procedures and the management of loaner instruments were revised in accordance with the latest available evidence. Feedback from association representatives was systematically collected and integrated into the final consensus recommendations. The consensus-building process was achieved by unanimous agreement among all delegates. This collaborative approach culminated in the development of three consensus documents aimed at addressing critical aspects of sterilization and reprocessing practices for surgical implants.

## Consensus recommendations

The consortium produced three documents focusing on different areas of sterilization and reprocessing. These addressed quality assurance in sterilization, standardized management of loaner and implantable devices, and the need for collaborative education and training initiatives to harmonize sterilization practices for surgical implants.

### Quality assurance strategies for sterilization monitoring

Quality assurance in sterilization is fundamental to the goal that all instruments used in patient care are free from contaminants. This is crucial for invasive procedures, where even minor lapses can result in severe complications for patients. As part of comprehensive quality assurance measures, sterilization indicators provide a systematic means of monitoring the effectiveness of sterilization processes and verifying the absence of microorganisms on medical equipment.^6,7^ These indicators objectively confirm whether key parameters such as temperature, pressure, and exposure time have been achieved. By promptly identifying process failures, sterilization indicators facilitate the removal of inadequately sterilized items from circulation, thereby minimizing infection risk. Furthermore, indicator results can be recorded in standard documentation practices for every sterilization cycle, supporting audits and maintaining a reliable, traceable history of instrument sterilization.^6,7^

**Figure 1.**
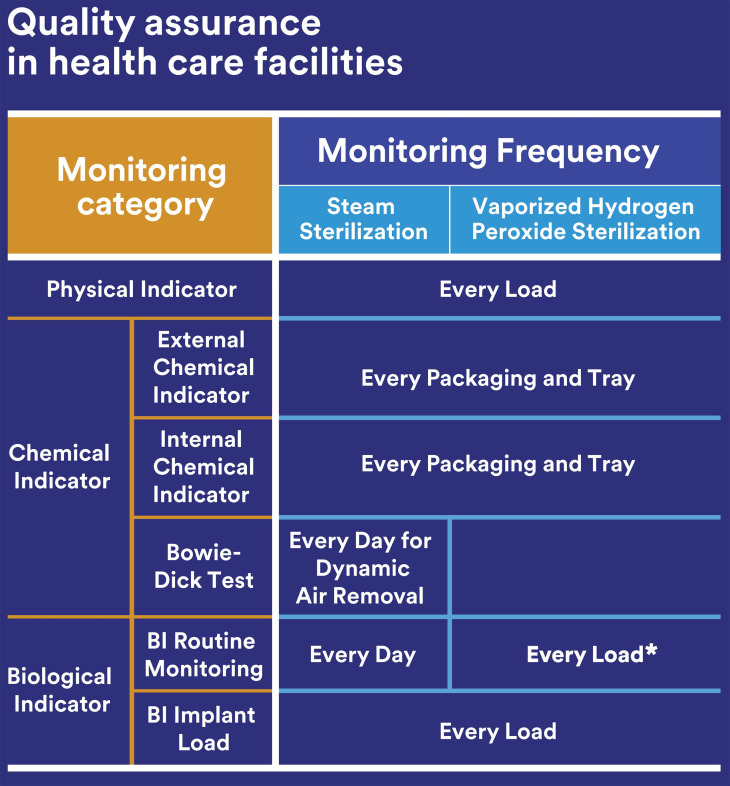
Medical device sterilization quality assurance in healthcare facilities. References: 1) Association for the Advancement of Medical Instrumentation. Comprehensive guide to steam sterilization and sterility assurance in health care facilities. Amendment 3. ANSI/AAMI ST79. 2) World Health Organization; Pan American Health Organization. Decontamination and reprocessing of medical devices for health-care facilities. World Health Organization; 2016. 3) Ling ML, Ching P, Widitaputra A, et al. APSIC guidelines for disinfection and sterilization of instruments in health care facilities. Antimicrob Resist Infect Control. 2018;7:25.

A concise overview of evidence-based quality assurance strategies for monitoring steam and hydrogen peroxide sterilization is shown in Figure 1.^6,8,9^ Employing a combination of physical, chemical, and biological indicators is essential for thoroughly evaluating the effectiveness and reliability of the sterilization process.^7,9^ Each indicator type monitors specific parameters, and their combined use ensures comprehensive oversight of all critical aspects. Relying on a single indicator may result in an incomplete assessment, whereas integrating multiple indicators introduces necessary redundancy, facilitates cross-verification, and enables the prompt identification of discrepancies or process failures. This multifaceted approach increases the likelihood of detecting sterilization issues, thereby ensuring that medical instruments and equipment are properly sterilized and free of harmful microorganisms. The recommended frequency of monitoring varies according to both the indicator type and the sterilization method employed.

Although strong evidence backs these recommendations, implementation is often hindered by resource limitations, staff shortages, workflow pressures, and differences in local infrastructure. Overcoming these barriers involves tailoring guidelines to local needs and investing in resources, training, and systemic support.

### Proper management of loaner instruments and implant

Loaner medical instruments and implants refer to surgical tools and devices that are provided on a temporary basis to healthcare facilities by external vendors or manufacturers.^10,11^ These items are not owned by the hospital but are lent for specific procedures, particularly when the institution does not possess the required equipment or when specialized implements are necessary for uncommon or complex surgeries, such as orthopedic or spinal operations. Improperly sterilized loaner medical instruments and implants pose significant risks to patient safety, healthcare operations, and overall infection control. Compared to hospital-owned equipment, these pose specific challenges to proper sterilization, including unfamiliarity with the devices and time constraints.^12^ It is for this reason that training and strict adherence to protocol are warranted.

**Figure 2.**
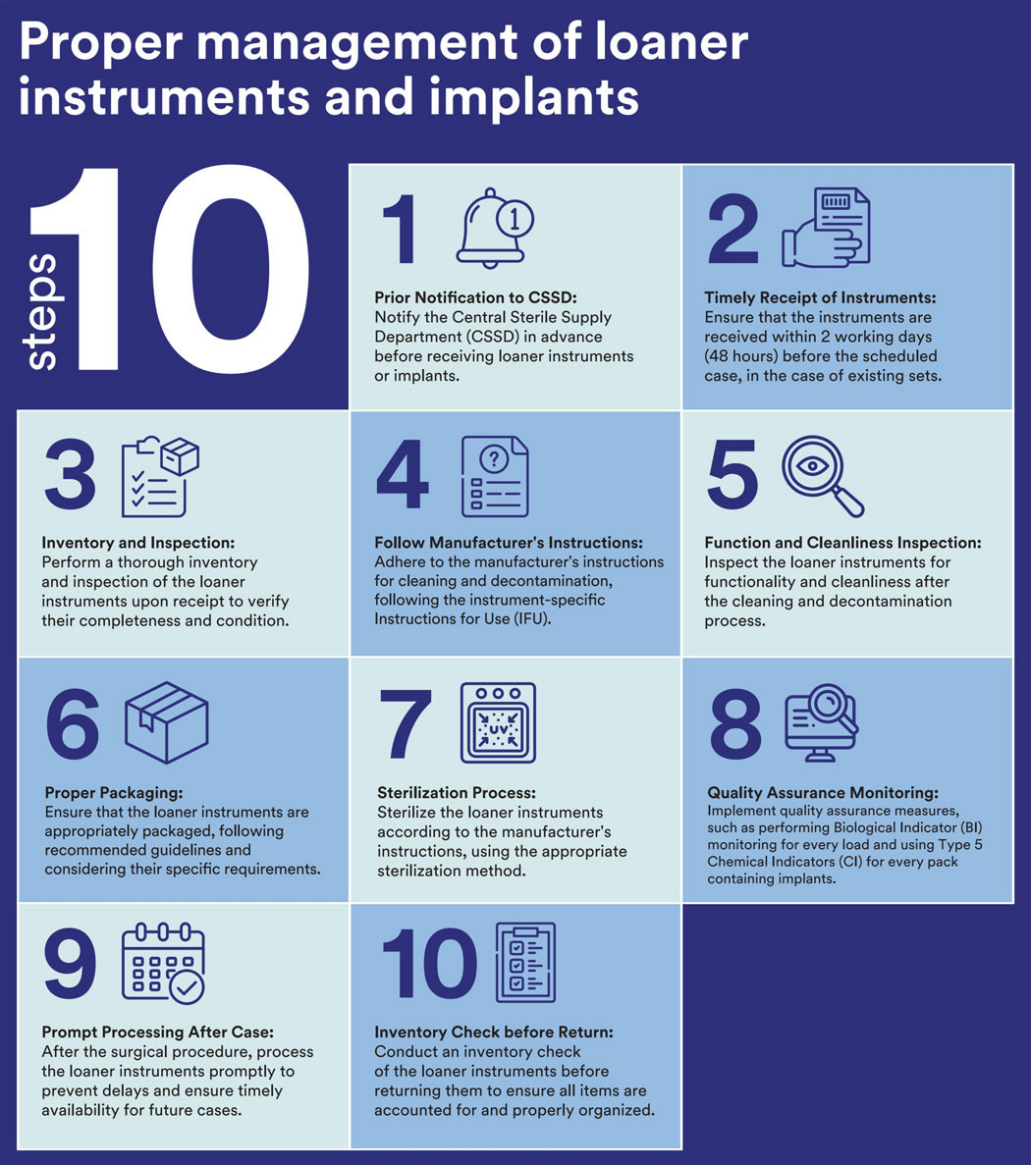
Steps for the proper management of loaner instruments and implants. References: 1) Association for the Advancement of Medical Instrumentation. Comprehensive guide to steam sterilization and sterility assurance in health care facilities. Amendment 3. ANSI/AAMI ST79. 2) World Health Organization; Pan American Health Organization. Decontamination and reprocessing of medical devices for health-care facilities. World Health Organization; 2016. 3) Ling ML, Ching P, Widitaputra A, et al. APSIC guidelines for disinfection and sterilization of instruments in health care facilities. Antimicrob Resist Infect Control. 2018;7:25. 4) Seavey R. Reducing the Risks Associated With Loaner Instrumentation and Implants. *AORN Journal*. 2010;92(3):322–334.

The consortium determined that 10 steps should be required for the proper management of loaner instruments and implants (Figure 2). These emphasize the importance of timely communication, thorough inspection, adherence to manufacturer guidelines, and systematic processing to ensure safety and efficiency in surgical settings.

Timeliness is a critical component of effective reprocessing, as hastening the process may increase the likelihood of errors, reduce availability, and result in missed procedures. Scheduling, planning, and timely communication enable effective processing and thorough checking and reduce the likelihood of mistakes.^11^ In many countries, it is not uncommon for loaner instruments to arrive less than 24 hours before the surgical procedure.^13^ Whenever possible, it is important for clinical staff to take measures to ensure that the instruments are received within 48 hours before the scheduled case.^10^ Likewise, loaner instruments should be promptly processed after use to ensure timely availability for future cases.

Proper notification and documentation of loaner medical devices and implants play a role in patient safety, regulatory compliance, inventory management, accountability, and quality control.^10^ These practices allow tracking device usage and history, which supports identification and removal in the event of recalls or issues. Adhering to health authority standards assists with compliance and may lower the risk of legal complications. Accurate records contribute to effective sterilization and reprocessing procedures, helping to reduce cross-contamination and infection risks. Inventory is managed more efficiently by monitoring devices for timely return and future availability. Documentation establishes an accountable record from manufacturer to patient, facilitating quality control and responses to adverse events. Comprehensive records also support financial processes such as billing and insurance claims by documenting costs and minimizing discrepancies.

Following instructions and protocols when managing loaner medical devices and implants is essential for ensuring patient safety, regulatory compliance, device integrity, traceability, and cost efficiency.^11^ Proper handling and maintenance reduce the risk of infections and complications, ensure compliance with healthcare regulations, maintain device functionality, and allow for accurate tracking and documentation. This not only helps in avoiding legal issues and potential fines but also ensures the availability of devices when needed, ultimately contributing to the overall efficiency and reliability of the healthcare system. By adhering to these protocols, healthcare professionals can provide the highest standards of care and maintain the trust of their patients and regulatory bodies.

### Sterilization recall policy and procedure

A medical device sterilization recall policy is a formal, written procedure that healthcare facilities use to respond when a sterilization process fails or is suspected to have failed. Its main purpose is to identify and address lapses in the sterilization process, thereby protecting patients from exposure to potentially contaminated medical devices.^6^ By ensuring that any sterilization failures are quickly detected and rectified, such policies uphold the integrity of the sterilization workflow and support high standards of care.

**Figure 3.**
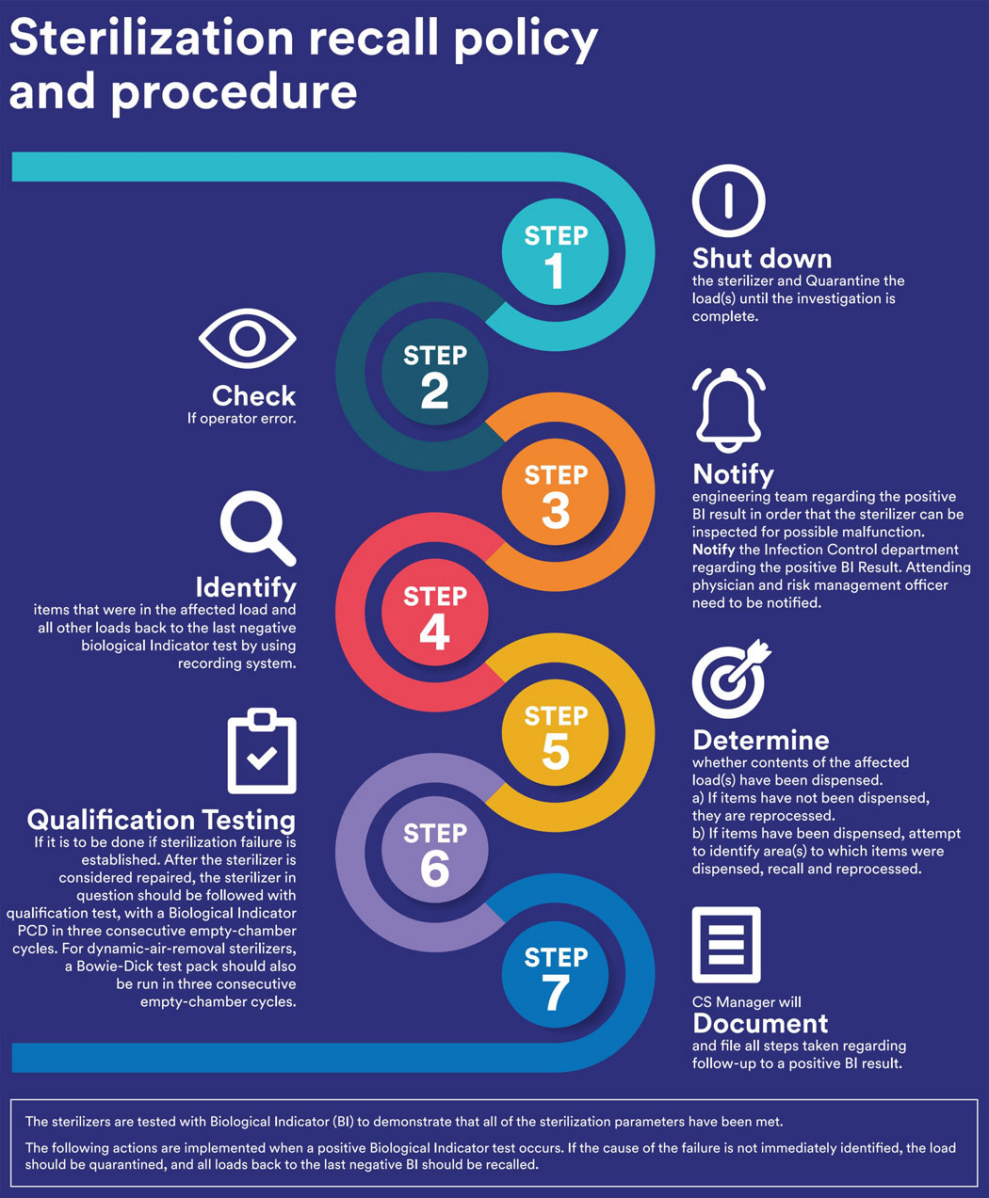
Sterilization recall policy and procedure. References: 1) Association for the Advancement of Medical Instrumentation. Comprehensive guide to steam sterilization and sterility assurance in health care facilities. Amendment 3. ANSI/AAMI ST79. 2) World Health Organization; Pan American Health Organization. Decontamination and reprocessing of medical devices for health-care facilities. World Health Organization; 2016. 3) Ling ML, Ching P, Widitaputra A, et al. APSIC guidelines for disinfection and sterilization of instruments in health care facilities. Antimicrob Resist Infect Control. 2018;7:25.

Well-defined recall policies support regulatory compliance and provide traceability for audits and investigations. Furthermore, they enable healthcare facilities to learn from incidents and improve their sterilization practices in the future. A general outline of an effective recall policy is shown in Figure 3. Each of the seven steps represents a critical component to systematically address potential sterilization failures and mitigate associated risks. Healthcare facilities that adopt these recall steps can improve patient safety by preventing patient exposure to contaminants during invasive procedures, proactively assessing clinical risk, supporting instrument traceability, and preparing to take corrective action, if needed.

Timely intervention is critical to prevent broader outbreaks of infection; therefore, operators must be equipped to recognize and isolate improperly sterilized loads, notify the Infection Control department, and initiate corrective procedures without delay.^14^ A quick response time helps minimize the adverse consequences (which may include medical, operational, and financial losses) associated with inadequately sterilized items. Once the root cause of the sterilization failure has been addressed, it is imperative to rigorously revalidate the process using biological indicators. This involves conducting multiple sterilization cycles and systematically testing the outcomes to verify that all required parameters are consistently achieved. Comprehensive documentation and monitoring of all findings, corrective actions, and validation results are vital to maintaining the ongoing effectiveness of the sterilization process.^14^

Healthcare facilities seeking to establish or improve recall protocols must overcome practical barriers, frequently in communication and documentation. Fragmented or informal channels can cause delays and inconsistencies that need to be navigated to enact timely recalls. Outdated or inconsistent record systems can hinder traceability, especially in high-volume or resource-limited settings. Ultimately, successful recall policies rely on institutional investment in clear lines of communication, defined interdepartmental roles, streamlined and integrated documentation, and regular training.

## Discussion

As shown by a recent survey conducted across Asia-Pacific countries, many current medical device sterilization practices fall short of recommended guidelines, such as insufficient monitoring and the absence of recall policies.^5^ These deficiencies underscore the necessity for regional collaboration and the development of consensus documents aimed at improving sterilization standards. Such recommendations can inform the creation of educational initiatives and provide a structured framework for policy enhancement.

Research indicates that healthcare professionals possess varying levels of understanding regarding sterilization techniques, underscoring the need for enhanced education and training. While many staff members recognize the importance of sterilization monitoring, consistent training and adherence to standardized procedures are often lacking. Major obstacles include high workloads, inadequate staffing, and restricted access to necessary resources, technology, and infrastructure. Addressing these challenges requires a comprehensive approach that encompasses investment in resources, alignment of regulatory standards, ongoing professional development, infrastructure improvements, heightened awareness, and the integration of advanced technological solutions.^15^

To address existing deficiencies and establish a framework for improvement within the Asia-Pacific region, the Asia Safe Surgical Implant Consortium developed three consensus documents to serve as foundational resources for CSSDs seeking to enhance their operational practices. These recommendations are grounded in internationally recognized guidelines as well as the collective expertise of the contributors, providing a robust basis for elevating sterilization standards across healthcare facilities.

The recommendations presented in this paper are strongly aligned with the guidelines set forth by key international and regional organizations, including the WHO, the Association for the Advancement of Medical Instrumentation (AAMI), and the Asia Pacific Society of Infection Control (APSIC). All three guidelines share similar emphasis on adherence to manufacturer instructions for use, routine monitoring, documentation, and training. The WHO guidance provides global, risk-based recommendations applicable across a broad range of reusable medical devices and healthcare settings, with particular attention to minimum safe practices and system-level processes.^9^ Similarly, the APSIC guidelines address sterilization practices while acknowledging the challenges specific to the Asia-Pacific region.^6^ In contrast, the consortium recommendations concentrate their focus on critical points of vulnerability within sterilization assurance, explicitly cautioning against reliance on a single monitoring modality, introducing structured, stepwise guidance for recall processes, and focusing on surgical implants and loaner instruments, a subset of critical devices associated with disproportionately high patient risk. Furthermore, this document is consistent with the seminal AAMI sterility assurance publication, ST79, which functions as a highly prescriptive technical standard optimized for well-resourced healthcare systems.^8^ The consensus-building process allows for greater contextualization of evidence-based practices within real-world operational environments, enhancing feasibility and relevance.

Achieving worldwide consistency in sterilization standards remains an ideal goal for healthcare systems, yet the practical realities make this a challenging objective. Resource constraints, regulatory discrepancies, and varying enforcement practices are major obstacles. Health-system heterogeneity, cultural variations, and even geography can influence clinical practices and can lead to uneven compliance. Therefore, the goal of guidance documents should be to align on core safety principles using flexible frameworks that ensure minimum patient safety requirements while allowing for local adaptation.

## Limitations

Consensus documents are shaped by expert opinion, which can introduce subjectivity and potential bias due to differing experiences, expertise, and possible conflicts of interest. As a result, the applicability of these recommendations may be limited when implemented across diverse healthcare settings. Furthermore, consensus documents may oversimplify the complexities inherent in clinical practice. To address these limitations, the panel incorporated the latest guidelines and the strongest available evidence. Additionally, representatives from various countries, disciplines, and institutions participated in the process to ensure a wide range of perspectives was considered.

## Conclusion

The expert panel’s initiatives produced consensus documents to provide practical guidance for improving quality assurance, device management, and recall procedures to enhance patient safety. Continued regional collaboration, resource investment, and professional development are crucial for achieving consistent adoption of best practices and safer surgical outcomes.
